# The Dark Side of IFN-γ: Its Role in Promoting Cancer Immunoevasion

**DOI:** 10.3390/ijms19010089

**Published:** 2017-12-28

**Authors:** Marija Mojic, Kazuyoshi Takeda, Yoshihiro Hayakawa

**Affiliations:** 1 Division of Pathogenic Biochemistry, Institute of Natural Medicine, University of Toyama, Sugitani 2630, Toyama 930-0194, Japan; marija.mojic@gmail.com; 2 Division of Cell Biology, Biomedical Research Center, Graduate School of Medicine, Juntendo University, Bunkyo-ku, Tokyo 113-8421, Japan; ktakeda@juntendo.ac.jp; 3 Department of Biofunctional Microbiota, Graduate School of Medicine, Juntendo University, Bunkyo-ku, Tokyo 113-8421, Japan

**Keywords:** IFN-γ, tumorigenesis, tumor immunogenicity, immune suppression

## Abstract

Interferon-γ (IFN-γ) is a pleiotropic cytokine that has long been praised as an important effector molecule of anti-tumor immunity, capable of suppressing tumor growth through various mechanisms. On the contrary to such a bright side of IFN-γ, it has also been involved in promoting an outgrowth of tumor cells with immunoevasive phenotype suggesting an existence of a dark “tumor-promoting” side effect of IFN-γ. In this review, we will summarize this multi-functional role of IFN-γ in tumor context, how it promotes changes in tumor phenotype towards increased fitness for growth in immunocompetent host. Furthermore, we summarize how IFN-γ is involved in homeostatic or cancer-triggered mechanisms to establish an immunosuppressive tumor microenvironment.

## 1. Background

Interferon-γ (IFN-γ) is a pluripotent cytokine discovered as part of a larger family of factors—interferons, named after their ability to interfere with the growth of live viruses [1]. It is a lone member of type II interferons that is produced by several innate and adaptive immune cells in response to dangerous stimuli. Thus, it is indispensable for physiological processes primarily related to immune regulation and anti-microbial/anti-viral host defense. Moreover, diverse biological actions of IFN-γ are also implicated in pregnancy, obesity, allergies, autoimmune diseases, as well as in cancer [2,3,4,5,6].

In cancer biology, results from early studies established IFN-γ as a prototypical antitumor cytokine. However, findings that endogenous IFN-γ not only controls tumor initiation and progression but also shapes tumor immunogenicity and promotes the outgrowth of tumor cells with immunoevasive properties [7,8,9] revealed that this cytokine can have a dual role in shaping cancer’s outcome. Here, we will review recent findings that unveil complex aspects of IFN-γ in its tumor-promoting effects. 

## 2. Interferon-γ (IFN-γ) Producers in Tumor Microenvironment

In a tumor microenvironment, numerous cells of innate and adaptive immunity produce IFN-γ. Two main producers of IFN-γ are natural killer (NK) cells, providing signals from their activating receptors that prevail over signals stemming from inhibitory receptors, and tumor-specific cytotoxic CD8^+^ T lymphocytes (CTLs) following antigen stimulation of T cell receptor (TCR) or in TCR-independent way that involves synergistic stimulation with cytokines IL-12 and IL-18 [10,11]. Th1 polarized CD4^+^ T helper cells, known for their ability to help in the promotion and maintenance of anti-tumor CTL responses, also secrete IFN-γ that recruits various cells of innate and adaptive immunity to tumor sites and promote their activation [12]. Immune cells that belong to a blurred distinction between innate and adaptive immunity also produce significant amounts of IFN-γ in tumor microenvironment. Such cells are natural killer T cells (NKT), which express αβ TCR and NK cell receptors, and innate-like γδ T cells, whose antigen recognition by γδ TCR- is not restricted to major histocompatibility complex (MHC) molecules [13,14]. Th22 IFN-γ^+^CD4^+^ T cells, macrophages, IFN-producing killer dendritic cells (IKDC) and group 1 innate lymphoid cells (ILC1) are some of the recently discovered immune cells found to infiltrate tumors and to produce IFN-γ [15,16,17,18]. With revived scientific interest for studying immune components of tumor microenvironments, the list of IFN-γ producers is expected to be further expanded.

## 3. IFN-γ Signaling Pathways

IFN-γ mediates its diverse biological effect by binding to IFN-γ receptor (IFNGR), expressed on the surface of nearly all types of cells. In canonical IFN-γ signaling, binding of IFN-γ to its receptor causes oligomerisation of receptor’s subunits IFNγR1 and IFNγR2 and activation of downstream receptor-associated Janus kinases, JAK1 and JAK2. If not inhibited by physiological negative regulator suppressor of cytokine signaling 1 (SOCS1), JAK1/2 phosphorylate and activate signal transducer and activator of transcription 1 (STAT1) in most cells and STAT3 in some cells. Activated homodimers, also known as γ interferon-activated factor (GAF), accumulate in the nucleus and act as transcription factors binding to γ interferon-activated sequence (GAS) elements present in promoters of most IFN-γ-inducible genes. A major primary response gene, trans-activated by canonical IFN-γ signaling, encode the interferon regulatory factor 1 (IRF1), a transcription factor that further activates a large number of secondary response genes. Co-operating with canonical the JAK-STAT signaling pathway, or acting parallel with it, other pathways that include MAP kinase, PI3K, JNK, CaMKII and NF-κB regulate many aspects of IFN-γ biological actions [19,20,21,22]. 

## 4. IFN-γ-Mediated Anti-Tumor Responses

Early studies of IFN-γ effects on various cancer types revealed its extensive anti-tumor potential. Among them, the best known IFN-γ-mediated effect is augmentation of cytotoxic function of NK cell and CTLs as potent effectors of anti-tumor responses [23]. It is also very well known that IFN-γ enhances antigenicity of tumor cells via up-regulation of the major histocompatibility complex (MHC) class Ia membrane expression. As tumor cells express antigens that differ from their non-transformed counterparts, e.g., neo-antigens resulting from gene mutations, overexpressed cellular antigens or viral antigens [24], IFN-γ stimulates expression of tumor antigen-presenting MHC molecules to increase immunogenicity of tumor cells and makes them more susceptible to immune recognition and destruction [25,26,27]. IFN-γ also displays direct anticancer activity via inhibition of cell proliferation, e.g., by upregulation of p21 and p27 molecules to arrest the cell cycle, or through mediation of apoptotic cell death [28,29,30]. Moreover, by targeting non-transformed cells present in the tumor microenvironment, IFN-γ displays its indirect anti-tumor actions, acting as an antiangiogenic factor to inhibit tumor angiogenesis and/or to promote destruction of established tumor-associated blood vessels [31,32,33,34].

In past key studies, IFN-γ has been shown to play a central role for sketching the outlines of immunoediting concept. This concept frames the transition of the immune system’s role from being tumor restrictive to tumor permissive through three sequential phases: elimination, equilibrium and the escape phase. During these phases, tumors are sculpted to be fit to grow in immunocompetent host [35]. In the absence of IFN-γ signaling, the initiation and progression of chemically induced, transplanted or spontaneously arising tumors in mice were facilitated [8]. Moreover, the importance of IFN-γ for immune destruction of tumors during the elimination phase and for maintenance of the equilibrium phase where occult tumor outgrowth is controlled by immunity has also been studied extensively [8,9,36,37]. Those series of studies in mice models that were either IFN-γ deficient or had endogenous IFN-γ blocked with monoclonal antibody clearly indicate the anti-tumor role of IFN-γ. Collectively, IFN-γ was shown to exert its potent anti-tumor effect through modulating immune cells, tumor cells and/or non-immune stromal cells in tumor microenvironment.

## 5. IFN-γ in Clinical Settings

Because of its potent anti-tumor activity, IFN-γ has been regarded as a promising cancer immunotherapy agent. Although IFN-γ was rarely tested as a single agent, most of earlier clinical trials examined the antitumor potential of recombinant IFN-γ (IFN-γ1b) as an adjuvant to surgery or conventional chemotherapy [38]. Overall, those clinical trials of IFN-γ had mixed success, often reporting conflicting outcomes in patients with the same tumor type. Promising results were obtained in a study where prophylactic treatment with IFN-γ prevented recurrence of bladder tumor after transurethral tumor resection [39]. Equally promising results IFN-γ1b used for treatment of patients with adult T cell leukemia have been reported capable of inducing lasting remissions [40], while administration of adenovirus vectors that express IFN-γ cDNA showed a clinical benefit for patients with cutaneous T and B cell lymphomas [41,42]. IFN-γ treatments led to upregulated human leukocyte antigen-D related (HLA-DR) expression, a human MHC class II molecule, and improved prognosis of colorectal patients [43]. However, another clinical study in which IFN-γ was used as an adjuvant therapy after surgical resection of colon cancer showed no therapeutic benefit [44]. Early clinical trials of IFN-γ treatment of melanoma went rather inconclusive in its efficacy, as a small number of patients was involved. Other trials of IFN-γ in melanoma as an adjuvant to conventional treatment had to be prematurely terminated, as patients that received IFN-γ had worse clinical outcomes than the IFN-γ-untreated patients [45,46,47,48,49]. In ovarian cancer, clinical trials of IFN-γ shared a similar fate to melanoma. Initial studies showed quite promising results and IFN-γ was used as a single agent in second-line treatment or included in the first-line platinum-based treatment and resulted in improved progression free survival of those patients [50,51]. In contrast, it is also reported that ovarian cancer patients treated with IFN-γ suffered from severe adverse effects and showed shorter overall survival rates [52]. Despite such inconsistent results of previous clinical studies, IFN-γ is still tested as a treatment for HER-2 positive breast cancer, ovarian cancer, fallopian tube cancer, primary peritoneal cancer and soft tissue sarcoma (ClinicalTrials.gov: NCT03112590, NCT02948426 and NCT03056599). Contrary to such inconsistent outcome of the clinical use of IFN-γ, it certainly plays an important role in the success of a new generation of immunotherapy, which has proved to be superior to other means of cancer management. A reinvigoration of anti-tumor immune responses by treating with immune checkpoint inhibitors is strongly accompanied with the increased serum levels of IFN-γ and the IFN-γ-induced chemokines (CXCL-9 and CXCL-10) together with increased numbers of IFN-γ-producing T cells in both peripheral blood and tumor tissues [53]. In fact, there is an ongoing clinical trial that is testing if addition of IFN-γ treatment to PD-1 inhibitor nivolumab immunotherapy of patients with advanced solid tumors will be well-tolerated and have additional beneficial effects (ClinicalTrials.gov: NCT02614456). Similar to immune checkpoint inhibitor treatment of cancer, IFN-γ is also a determinant of efficacy of adoptively transferred T cell therapies [54,55]. 

## 6. Initial Discovery of Tumor Promoting Features of IFN-γ

**Figure 1:**
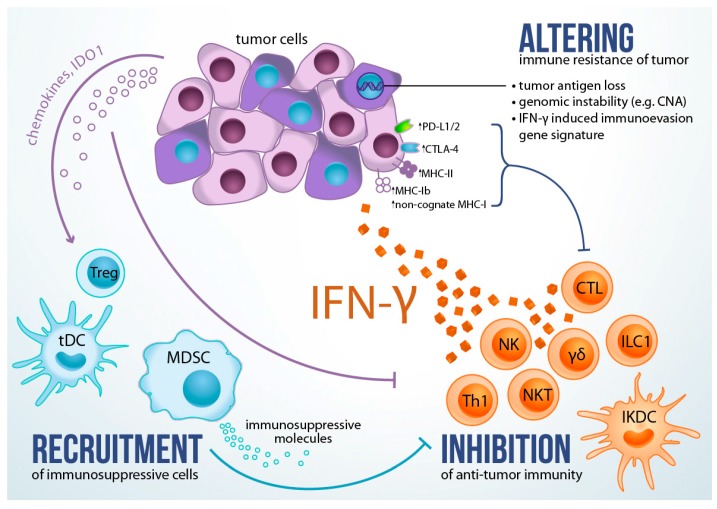
IFN-γ in promoting tumor immunoevasion. In response to immunogenic cancer, cells of anti-tumor immunity secrete IFN-γ (orange cells), which in turn induces genomic instability (e.g., copy number alterations, CNA) and/or immunoevasive gene expression signature in cancer cells (PD-L1, PD-L2, CTLA-4, non-classical MHC class Ib antigens, IDO1 etc.), sculpting tumor cell phenotype towards increased fitness for growth in immunocompetent host. In parallel, IFN-γ establishes immunosuppressive tumor microenvironment by triggering homeostatic response to limit inflammation, promoting tumor cells to produce immunosuppressive molecules (e.g., IDO1 that causes local depletion of amino acid tryptophan suppressing CTL and NK cells and activating Tregs and MDSC), or recruiting immunosuppressive cells (blue cells). CTL, cytotoxic CD8^+^ T cells; NK, natural killer cells; γδ, γδ T cells; NKT, natural killer T cells; Th1, Th1 polarized CD4^+^ T helper cells; ILC1, group 1 innate lymphoid cells; IKDC, IFN-producing killer dendritic cells; tDC, tolerogenic dendritic cells; Treg, regulatory T cells; MDSC, myeloid derived suppressor cells; MHC-I, major histocompatibility complex molecule class I; MHC-II, major histocompatibility complex molecule class II; CNA, copy-number alterations; IDO1, indoleamin-2,3-dioxygenase 1, a tryptophan-metabolizing enzyme.

About the same time when IFN-γ was galloping toward fame as a multitasking anti-tumor agent, first reports of its tumor promoting actions started to appear. Taniguchi et al. showed that IFN-γ pre-treatment potentiated lung colonization of intravenously inoculated B16 melanoma due to upregulation of MHC class I molecules H-2Kb and H-2Db on tumor cells and decreased sensitivity to NK cells [56]. Greater metastatic potential in vivo, also attributed to increased resistance to NK cells, was observed in IFN-γ-gene-transfected TS/A mammary adenocarcinoma cells [57]. Another study reported that IFN-γ treatment of human melanoma cells in vitro resulted in favorable growth inhibition, but also increased expression of several markers expressed in advanced melanomas (e.g., HLA-DR and -DQ, ICAM-1 and A.1.43), suggesting the potential of IFN-γ to promote the development of more aggressive phenotypes in cancer cells [58]. These first clues from pre-clinical research of pro-tumorigenic features of IFN-γ, coupled with occasional reports from clinical studies of tumor pro-growth effects of recombinant IFN-γ, strongly suggested the existence of a double-sided role of IFN-γ in tumor control. As a result of extensive studies over the past three decades, it is now evident that first, IFN-γ can facilitate tumor initiation and, subsequently, promote changes in tumor cell phenotype towards increased fitness for growth in immunocompetent host; and second, that it is intimately involved in several homeostatic or cancer-triggered mechanisms that promote establishment of immunosuppressive tumor microenvironment (Figure 1).

## 7. Role of IFN-γ in Promotion of Tumor Growth 

Chronic inflammation is known to contribute to all stages of tumorigenesis, from tumor initiation and promotion to progression. Many cytokines produced in chronic inflammation, such as TNF-α and IL1-β, are already reported to fuel cancer growth and progression toward more malignant phenotypes through multiple mechanisms including an induction of DNA damage response, angiogenesis, and activation of signaling pathways that promote cancer cell survival and/or proliferation [59,60]. Several studies have outlined the role of chronic IFN-γ in tumorigenesis. Yoshida et al. showed that development of carcinogen-induced hepatocellular carcinomas is enhanced in mice with a heterozygous deletion of the *SOCS1* gene [61]. Besides being a negative regulator of IFN-γ signaling, SOCS1 is known to inhibit cytokine signaling mediated by JAK-STAT pathways (e.g., IL-4 and IL-6) and Toll-like receptor (TLR) signaling [62]. Thus, contribution of factors other than IFN-γ in the SOSC1^−/−^ tumorigenesis model cannot be excluded. Using SOCS1^−/−^ transgenic (Tg) mice, in which exogenous SOCS1 expression was restored only in T and B cells on a SOCS1^−/−^ background, Hanada et al. found that constitutive IFN-γ-dependent STAT1 signaling is necessary for spontaneous development of colon cancer, which can be prevented with anti-IFN-γ antibody treatment [63]. Recently, Zou et al. showed that excessive and chronic IFN-γ production by CD4^+^ T cells, caused by T cell intrinsic deficiency for deubiquitinase USP15, increases incidence of methylcholantrene (MCA)-induced fibrosarcomas. Contrary to the role of chronic IFN-γ in MCA-induced carcinogenesis, the growth of the transplantable B16 tumor was significantly suppressed by IFN-γ in the same animal model, and these discrepancies could be attributed to the variations in duration of IFN-γ exposure (acute versus chronic), and quite possibly to the differences in tumor types [64]. In other in vivo transplantable cancer models, chronic exposure to low levels of IFN-γ has been reported to promote growth of H22 hepatoma, MA782/5S mammary adenocarcinoma and B16 melanoma [65]. Moreover, Zaidi et al. showed that IFN-γ signaling can trigger initiation, survival and/or outgrowth of UVB-induced melanoma cells [18]. IFN-γ secreted from adoptively transferred CTLs was found to promote proliferation of leukemia stem cells (LSC) and leukemia progenitor cells in a mouse model of chronic myeloid leukemia. Interestingly, LSC expansion in this model was observed only when high amounts of IFN-γ were secreted [66]. Collectively, chronic exposure to IFN-γ can promote tumorigenesis, however, there might be an undefined threshold in the level of IFN-γ exposure to regulate this process and/or it could vary depending on the tumor cell type. 

It has been widely recognized that tumors induced by MCA in mice deficient in the IFN-γ pathway or treated with anti-IFN-γ antibody show higher immunogenicity compared to those inimmunocompetent mice [36,37]. From those evidences, IFN-γ selects tumor cells capable of evading immune responses possibly through a constant selective immune pressure. Such immune-evaded tumor cells are frequently found to lose their tumor antigen expression, which can be a result of reduced expression levels of tumor antigens, loss of MHC class I expression, or impaired antigen processing machinery in tumor cells [67,68]. It was reported that IFN-γ exposure led to downregulation of endogenous tumor antigen expression in M14 melanoma and CT26 colon cancer cell lines [69,70]. It is also reported that human melanoma cell lines treated with IFN-γ for prolonged periods lost melan-A and gp100 processing and subsequently evaded CTL recognition [71]. In addition to classical MHC class I regulation, IFN-γ induced expression of non-cognate MHC-I molecules on mouse melanoma cells, which in turn limited tumor-antigen specific T-cell activation and effector functions [72].

## 8. Role of IFN-γ in Altering Immune Resistance of Tumor

In its simplest definition, tumor immunogenicity is the ability of a tumor to induce an immune response that can prevent its growth [73]. It is accepted that highly immunogenic tumors are the ones that express and present adequate levels of tumor-unique antigens, in a form that activates anti-tumor immunity instead of immune tolerance [73]. Aside from that earlier mentioned selection of poorly immunogenic tumor cell variants by IFN-γ-dependent immune responses, we recently reported that IFN-γ also induces genetic instability of tumor cells that corresponds to immune escape (Figure 1). As a result of in vivo exposure of tumors expressing immunogenic antigens to IFN-γ producing CTLs, we observed copy-number alterations (CNA) associated with DNA damage response and modulation of DNA editing/repair gene expression, which was not only attributable to the tumor-antigen loss but also accumulated during immunological selection thereby contributing to intra-tumor heterogeneity [74]. In addition to tumor-specific antigen loss, IFN-γ-induced genomic instability could result in an acquisition of beneficial phenotype alteration for tumor cells such as acquiring growth advantage, increasing metastatic potential and/or therapeutic resistance [75].

In addition to the reduction of their immunogenicity, tumor cells can evade IFN-γ-dependent immune responses by altering their IFN-γ signaling pathway (e.g., via downregulation or loss of IFN-γ receptor/downstream signaling molecules or through amplification of IFN-γ pathway inhibitors). First indication of such possibility came from the study of the MCA-induced tumorigenesis model in which IFN-γ receptor deficient mice developed tumors more rapidly and with higher incidence than those of IFN-γ-responsive counterparts [8]. Loss of the IFN-γ receptor was found to predict poor prognosis in ovarian cancer and was also seen as an underlying reason for the limited success in the therapeutic use of IFN-γ in ovarian cancer trials [76]. Recently, two independent reports indicated the importance of IFN-γ responsiveness and/or signaling for acquiring tumor cell resistance against immunotherapy [77,78]. In addition, Patel et al. also found that loss of expression of genes that have key roles in IFN-γ signaling, as well as in antigen presentation, serve as a main mechanisms of tumor immune evasion and are underlying causes for cancer resistance/non-responsiveness to immunotherapies by using “two cell type” (2CT)-CRISPR assay [78]. Importantly, the clinical analysis of tumors in patients who relapsed or did not respond at the first place to immune checkpoint inhibitors revealed genomic alterations resulting in loss of IFN-γ signaling-related genes (*IFNGR1*, *IRF1*, *JAK1*, *JAK2*, *IFNGR2*) and amplification of IFN-γ pathway suppressor genes (e.g., *SOCS1*, *PIAS4*) [79,80,81]. Thus, responsiveness to IFN-γ can be a critical determinant for the fate of cancer cells in response to anti-tumor immunity and immunotherapy.

## 9. Role of IFN-γ in Promoting Immunosuppressive Tumor Microenvironment

IFN-γ has also been known to induce many homeostatic pathways that limit tissue destructions and facilitate recoveries upon resolution of inflammation. There is mounting evidence that malignant tumor cells are taking advantage of such homeostatic features of IFN-γ to attenuate antitumor immunity and boost their own progression. Amongst more than 200 genes induced by IFN-γ [82], many of those genes are the molecules involved in cancer cell immune evasion, such as *PD-L1*, *PD-L2*, *CTLA-4*, *CIITA*, non-classical MHC class Ib antigens, *IDO1*, *CXCL12* etc. [18,19,83]. IFN-γ promotes expression of PD-L1 and PD-L2 not only in tumor cells, but also in other stromal cells including immune infiltrating cells, and suppresses the effector function of tumor-specific T cells or NK cells through an interaction with an immune inhibitory receptor, PD-1 [84,85,86]. IFN-γ induced PD-L1/2 expression has also been referred to as a mechanism of adaptive immune resistance to immune checkpoint therapy [87]. Moreover, Benci et al. showed that prolonged IFN-γ signaling in tumor cells triggers STAT-1-dependent epigenetic and transcriptional changes of tumor cells. As a consequence of those changes, expression of multiple ligands for T cell inhibitory receptors are induced in tumor cells which in turn promotes their PD-L1-independent resistance to immune checkpoint therapy [88]. In addition to the role of PD-1/PD-1ligands in downregulation of immune responses (Figure 1), tumor cell intrinsic PD-L1 was found to support tumor-initiating cells that are known to be resistant to conventional anti-tumor drugs and to cause a relapse of tumor [89]. In addition to the PD-1molecule, CTLA-4 is a rather classic inhibitory receptor that functions as an immune checkpoint molecule facilitating immune evasion through induction of tolerance to cognate antigens. Mo et al. recently reported that IFN-γ enhances CTLA-4 expression on melanoma cells which causes immune evasion [90]. MHC class I and IFN-γ also upregulate the expression of MHC class II trans-activator CIITA in melanoma cells leading to the upregulation of MHC class II antigen presentation that is associated with malignant progression and resistance to apoptosis [91,92,93,94]. Non-classical MHC class Ib molecules, e.g., HLA-E, HLA-F and HLA-G, are known to promote tumor escape from CTL and NK cell immune surveillance by binding to their inhibitory receptors (Figure 1). Indeed, Derre et al. showed that IFN-γ increased surface expression and shedding of soluble HLA-E molecules in melanoma cell lines, resulting in decreased susceptibility of tumor cells to CTL lysis [95].

Besides the induction of ligands for negative receptors of anti-tumor immunity, tumor cells often produce immunosuppressive molecules and/or can induce stromal cells with immune-suppressive character (Figure 1). The immunoregulatory tryptophan-metabolizing enzymes indoleamin-2,3-dioxygenase (IDO) 1 and 2, are known to induce CD4^+^CD25^+^Foxp3^+^ regulatory T cells (Treg), which is a key player of suppressing anti-tumor immune responses [96]. It was shown that IFN-γ promotes the expression of IDO in tumor cells, which consequently inhibits CTL-mediated anti-tumor response through Treg-dependent immune suppression [86,97]. Increased IDO1 expression was also found in hypermutated colorectal cancers, but not in non-hypermutated cancers, suggesting its role in blunting immune responses elicited against neoepitopes in these tumors [98]. Another potent suppressor of antitumor immune response is myeloid-derived suppressor cells (MDSCs). MDSCs are known to produce immunosuppressive cytokines, metabolically obstruct CTL responses by sequestering arginine, tryptophan and cysteine, and promote Treg induction and infiltration to tumor microenvironments [99]. The presence of MDSCs within tumors was shown to be IFN-γ-dependent to suppress anti-tumor effector T cell responses [99]. It has been reported that the IDO expression by tumors mediates expansion, recruitment, and activation of MDSCs in a Treg-dependent manner [100]. Moreover, excessive IFN-γ production by USP15-deficient CD4^+^ T cells promoted the expression of CXCL12 leading to an accumulation of T-bet^+^ Treg cells and CD11b^+^Gr-1^+^ MDSC, therefore promoting MCA-induced tumorigenesis [64]. Dendritic cells (DC) are important for shifting polarization of T cell-mediated immune responses, as they can prime both tumor-specific CTLs and suppressor regulatory T cells [101], thus regulating a homeostatic balance between immune responses and protective tolerance induction. Interestingly, IFN-γ promotes conversion of immunogenic DCs into IDO^+^ tolerogenic DCs by inducing IDO1 expression, thus limiting the extent of anti-tumor immunity [102,103]. In addition, it was recently reported that IFN-γ plays an important role to induce homeostatic gene programs in different mononuclear phagocytes including dendritic cells, monocytes and macrophages [104]. IFN-γ promoted self-tolerance during differentiation and migration of phagocytes into and/or from peripheral tissue, and the same IFN-γ-dependent-programs were enriched in the myeloid compartment of primary human tumors [104].

## 10. Concluding Remarks

Success in recent immunotherapies of cancer, autologous tumor-infiltrating lymphocytes (TIL)/chimeric antigen receptor (CAR) adoptive transfer or re-invigoration of anti-tumor immunity with immune-checkpoint inhibitors, is largely attributable to IFN-γ-dependent anti-tumor effects. However, a large proportion of patients show primary resistance to those treatments, and further immunotherapy relapsing cases are starting to emerge. Within immunogenic or inflammatory tumor microenvironments, a chronic presence of IFN-γ fails to contribute to tumor cell eradication by its “bright side” function, and instead, can induce a selection and/or generation of tumor clones with more malignant phenotype by its “dark side” function. Genetic screening of tumor cells recently revealed a pivotal role of tumor IFN-γ response for sensitivity to immunotherapies [77,78]. Besides, this pathway will also lead to an acquired resistance character against immunotherapies. Therefore, we have not averted our eyes from the “dark side” and confront this crafty trap. Several studies propose IFN-γ blockade as an approach for disruption of immunosuppressive tumor microenvironments or for suppression of IFN-γ-induced epigenomic and transcriptomic changes in tumor cells that provide a molecular basis for their immune escape [64,88]. Nevertheless, this approach may require further optimization as premature IFN-γ blocking might interfere to generate an effective anti-tumor response. Extensive future work will be needed to understand such complex roles of IFN-γ in tumor microenvironments and the full context of either pro- or anti-tumor features of IFN-γ. By enhancing its beneficial anti-tumor effects along with limiting pro-tumor actions, IFN-γ could contribute in establishing promising immunotherapy of cancer, and further reduce risks of tumors acquiring resistance to anti-tumor immunity and/or developing a progressive character.
